# Validity and Reliability of “Parental Attitudes of Various Aspects of Cochlear Implantation” Questionnaire

**Published:** 2015-11

**Authors:** Simin Soleimanifar, Zahra Jafari, Masoud Motasaddi Zarandy

**Affiliations:** 1 *Department of Audiology, School of Rehabilitation Sciences, Iran University of Medical Sciences (IUMS), Tehran, Iran.*; 2 *Department of Basic Sciences in Rehabilitation, School of Rehabilitation Sciences, Iran University of Medical Sciences (IUMS), Tehran, Iran.*; 3 *Otorhinolaryngology Research Center, Tehran University of Medical Sciences, Tehran, Iran.*

**Keywords:** Attitudes, Children, Cochlear implant, Parent, Questionnaire, Reliability, Validity

## Abstract

**Introduction:**

Parents are such important members of the cochlear-implant team that analysis of their views is essential in order to improve services and outcomes. The authors developed a tool to assess parental attitudes towards various aspects of cochlear implantation in children who had passed aural rehabilitation sessions. The authors then went on to determine the validity and reliability of this questionnaire.

**Materials and Methods:**

A questionnaire entitled, “Parental attitudes towards various aspects of cochlear implantation”, was prepared and assessed for content validity by experts in the field. The questionnaire comprised six subgroups, each scored using a five-point Likert scale. Parents of children with severe-to-profound congenital hearing loss who had undergone an aural rehabilitation program between 2007 and 2012 were eligible to take part in the questionnaire validation study (n=92, mean age of cochlear implantation 3.97 years). Test-retest reliability was subsequently assessed in 17 patients within 1 month.

**Results:**

The content validity index of the questionnaire was 98.68%.The external and internal reliability of the questionnaire was assessed using Cronbach’s alpha (0.844 and 0.892, respectively). Mean scores of the six subgroups of the questionnaire, including communication skills, academic skills, social skills, cochlear-implant center services, costs of surgery and rehabilitation programs and decision-making process and total were 84.6%, 75.0%, 84.0%, 78.8%, 83.4%, 67.0% and 79.2%, respectively.

**Conclusion:**

Overall, the results supported the validity, reliability and sensitivity of the questionnaire for use both in centers for cochlear implantation or aural rehabilitation clinics. The questionnaire would provide a valuable means of assessing the impact of cochlear implantation on children’s lives.

## Introduction

Delays in the diagnosis of severe-to-profound sensorineural hearing loss (SNHL) and the initiation of rehabilitation programs in children has an adverse effect on language acquisition, restricting children’s academic achievement and social opportunities (1,2). Studies over the past two decades have shown cochlear-implant usage to be an effective approach to rehabilitation in individuals with severe-to-profound hearing loss, unable to use traditional amplification such as hearing aids (3). Cochlear implantation technology has had a significant impact on the social integration, activities and confidence of children with low residual hearing. Cochlear-implant surgery allowed such children to benefit from the opportunity to learn languages and to attend regular schools (2,3). However, the degree to which this could be achieved was dependent upon several factors; such as period of sensory deprivation, general development potential, age at surgery and the degree of involvement by the child’s family (3).

Most studies of cochlear implantation (CI) devices to date have focused on their therapeutic impact on, for example, children’s ability to successfully acquire their first language (3). Few studies in the literature have so far addressed parents’ views on their child’s level of functioning post-CI (4). Attitudes, either positive or negative (5,6), are an evaluation of people, things, situations, events or activities and can be formed from a person’s past and present experiences (5). Attitudes ultimately consist of expectations, beliefs or emotional responses towards a person’s environment since this is a hypothetical construct that cannot be observed directly (7), objective measurement of attitudes can be hard to achieve (8). As important members of the CI team, parents’ views and attitudes are of critical importance to optimize effective service promotion and positive health outcomes. According to the growing prevalence of pediatric CI during past two decades, parental expectations have been raised. To date, little research has been carried into the influence of cochlear implants on physical, emotional and social functionality and acceptance among hearing peers (4). Problems in these areas might lead affected children to participate fewer in social activities, form fewer relationships and contribute to a sense of isolation. Some well-known questionnaires used to assess parental views following their child’s CI include Incesulu et al.’s, “Children with Cochlear Implantation Parental Perspectives Questionnaire” (CCIPP) and Archbold et al.’s closed-format questionnaire (1,9). The CCIPP included 74 items evaluating the quality of life of children post-cochlear implant, the services offered by CI centers as well as other aspects such as cost implications and support (1). Few studies have discussed children’s performance after receiving cochlear implants (4,10). Research has shown that increased parental awareness of the needs of their children post-cochlear implant could both accelerate and optimize rehabilitation goals (4,10).

During the course of the past decade, several thousand CI operations have been carried out in Iran, but no studies to date have investigated parents’ attitudes to their children having this procedure. The development of an accurate tool for evaluating parents’ views of cochlear implants could inform the planning of rehabilitation programs and parental education strategies. Questionnaires have been shown to be useful methods of studying parents’ views, providing valuable information about their awareness of their children's performance in various areas after CI surgery prosthesis (3). Parents have high expectations of the outcome following their child’s CI surgery, which itself places emotional and financial stress on the family. According to various methods of social and communication skills and also presence of cultural background within society across the world, it was necessary to evaluate implant outcomes and efficacy (3,4). In this study, the authors designed a questionnaire to evaluate outcome in children following CI surgery. Furthermore, this research aimed to test the validity and reliability of a questionnaire designed to assess parents’ attitudes to their children’s cochlear implants. 

## Materials and Methods

*Participants*

Ninety-two parents (47 of female and 45 of male children) of children with severe-to-profound SNHL took part in the study. Children in the study had undergone unilateral nucleus CI surgery at either the Amir Alam Cochlear Implant Center or the Rasool-E-Akram Hospital in Tehran between 2007 and 2012. All the children taking part in the study had attended rehabilitation classes for at least 1 year after CI surgery. No child in the study had any additional disabilities such as attention deficit, learning disabilities or developmental delay according their medical records. Suitable participants were selected from the Cochlear Implant Centers’ database by assessing a medical history form as completed by parents. Data collected included audiological and medical assessments (date of birth, medical history, demographic information). This study was approved by the Research Committee of the Iranian University of Medical Sciences.

*Procedure*

This study consisted of four main steps, which included preparation of test content, validity testing, implementation of research questionnaires and reliability testing.

*Test content*

The initial data-gathering phase of the study involved collection of background information such as family demographics, socioeconomic profile (i.e. educational level and economic status) and the child’s audiological history (including age at hearing loss and age at CI surgery). In order to analyze parents’ views on the outcome of CI surgery, the authors developed a detailed questionnaire, entitled “Parental attitudes of various aspects of cochlear implantation” (PAVACI), containing six fields addressing Iranian culture and life style. There were five response options (Likert scale) for each question, where 5=exactly, 4=good, 3= average, 2=somewhat and 1=none (11,12).

*Validity*

The initial questionnaire comprised 62 statements, whose contents were then validated by experts in the field. Each statement was scored on a four-point scale (1= not meeting selected criteria, 2=some modifications needed, 3=minor modifications needed, 4=meeting all selected criteria). The final questionnaire included 70 items, divided into six subscales: communication (11items); academic (9 items); social skills (15 items); social (15 items); Cochlear Implant Centers’ services (20 items), rehabilitation programs (5 items) and decision-making processes (10 items). Face validity of the questionnaire was also determined by asking 10 parents to assess how easily the questionnaire could be understood and undertaken (13,14).

*Implementation*

The questionnaire together with instructions as to how to complete and return it was given to parents in centers for CI surgery. The questionnaire was distributed by the researcher to the parents of children who had taken part in an aural rehabilitation program in a Cochlear Implant Centers for a minimum 1 year. A minimum of 1 year was considered appropriate to allow parents to assess the impact of CI surgery. Parents were informed that participation in the study was voluntary and that any decision not to take part would not affect their child’s care. 

*Reliability*

A test-retest analysis on 17 questionnaires was carried out at 2 weeks to determine the questionnaire’s reliability. . Internal and external reliability was calculated using Cronbach's alpha test (15).

*Data* *Analysis*

Data analysis was performed using SPSS 17.0, with p-values of ≤0.05 considered to be statistically significant. In this study, the content validity ratio (CVR) was calculated by the following formula for measuring the test validation (13,14).

$\mathrm{CVR}=\frac{E-N/2}{N/2}$

 ‘N’ refers to the numbers of experts and ‘E’ refers to the numbers of 3&4 option items chosen by the experts.

To obtain internal and external reliability, the intra-class correlation coefficient (ICC) was calculated and Cronbach's alpha was determined (15). Preliminary analysis with Kolmogorov-Smirnov tests showed an abnormal distribution of data (P<0.05). Significant relationship between age and total score, CI usage and total scores were analyzed using Spearman’s correlation test. 

To study the gender and age (pre-school and school age) effect, nonparametric Mann-Whitney U test was employed. 

## Results

*Demographics: *The average ages of children at the time of this study and receiving CI device were 7.18(±1.65) and 3.97(±1.89) years, respectively. In the present sample, all children had been amplified with right unilateral CI devices.

*Validity and Reliability: *The content validity index of the test was 98.68%. The sample contained 92 completed questionnaires, of which 17 were included in the test-retest analysis. In the external and internal reliability calculation, Cronbach's alpha equaled 0.844 and 0.892, respectively. A significant correlation between test and retest scores was demonstrated through a nonparametric Wilcoxon test (P=0.04). 

*Questionnaire*

 Analysis indicated that the average total score of parental attitudes was 79.2% (±0.22). Detailed information about each subscale score is presented in (Table.1).

**Table 1:** Descriptive statistics of all questionnaire subscales in children with cochlear implantation

**Mean Scores (and ±SD) with the parental questionnaire**								
	n	Communication skills (/11)	Academic skills (/9)	Social skills (/15)	Cochlear implant centers’ services (/20)	Costs of surgery and rehabilitation programs (/5)	Making decision process (/10)	Total (/70)
Children with Cochlear Implantation	92	84.6% (±5.4)	75.0% (±9.4)	84.0% (±.29)	78.8% (±5.8)	83.4% (±9.0)	67.0% (±6.8)	79.2% (±4.4)


As can be seen in Table.1, the highest score was related to communication skills (84.6%±5.4%) and the lowest to the decision-making process (67% ± 6.8%). There was a negative correlation between the total score average and the age at CI surgery (r=-0.21, P=0.04). Significant correlations were observed between the age at CI surgery and first and second subscales (r=-0.2, P=0.04 and r=-0.26, P=0.03, respectively).

Spearman’s correlation test indicated there was no significant correlation between the total score and average age at the time of this study (r=0.04, P=0.69). In the present study 51.1% percent of the population was female and gender had no effect on the results (P≥0.11).

## Discussion

 Validity of a study indicates that it has reflected or assessed the specific concept that the researcher was attempting to measure. In this instance, face validity was concerned with how a measure was viewed by parents (16-18). Before determining the final form of the questionnaire, 10 parents assessed it for ease of comprehension. A qualitative approach was used, whereby researchers collected the views of parents as to the intelligibility of the questionnaire to create an optimized set of questions. Content validity is based on the extent to which a measurement is shown to reflect the specific intended domain of content (16-18). One of the common methods used to assess this is to calculate the content validity index (CVI) (13,14,16). Using this method, the questionnaire developed in this study was found to have a content validity of greater than 0.75 (13,14); thus showing a high degree of content validity. Reliability is said to be the extent to which an experiment or any measuring procedure is shown to yield the same result on repeated trials. For a test to show external reliability, the scores should be the same or similar, even if that same test were taken several times at different intervals (16). Internal reliability of consistency is the extent to which tests or procedures are shown to assess the same characteristic, skill or quality. This reflects the inter-observer accuracy or the sensitivity of the measuring instruments used in a study. This type of reliability is useful in enabling researchers to interpret data, predict results and limit the relationship between variables (16,19). The external reliability of the study (ICC measure) was found to be 0.844, calculated using Cronbach’s alpha (16). Our results demonstrated that participants’ scores obtained during two consecutive trials 2 weeks apart were very similar to each other, showing the questionnaire to have a high level of external reliability. The internal reliability of the questionnaire was calculated as 0.892 indicating a high degree of internal consistency. According to Nunnally et al. (1978), a Cronbach’s alpha ICC score of at least 0.7 is consistent with a high level of internal consistency (16,18). Our study revealed that the questionnaire developed had a high level of reliability and reproducibility. 

Differences in cultures and healthcare systems may mean that quality of life is affected differently after CI surgery depending upon where the child is located (13). Therefore, it was necessary to create a questionnaire that could be applicable to many nationalities, cultural backgrounds and methods of communication (13).

In the present analysis of parents’ answers to the questionnaire, it was found that the highest level of parental satisfaction was seen in communication skills and the lowest in academic skills. The majority of parents were found to believe that the communication skills of children having had CI surgery were much higher than those achieved using hearing aids. The main reason for this was lack of speech clarity, leading to parental discontent. Many studies have found significant positive changes in the communication skills of hearing-impaired children following CI surgery. A direct link between the success of oral communication and other variables such as quality of life is also documented in the research literature (4,10,20). Incesulu et al. (2003) reported that family satisfaction with communication skill development in children was higher than other areas (1). Another study by Huttunen et al. (2009) in 36 Finnish children and their families revealed that the highest parental satisfaction was in the area of social and communication skills (2). The lowest score in this study was obtained on the decision-making process subscale. Our study revealed that most parents reported inadequate emotional and psychological support from the specialist CI center staff to assist their decision-making process. Scores obtained in the CI centers’ services and decision-making subscales in our study were consistent with those in the similar Lutermanand Kurtzer-White and Wong report. Researchers noted that parents’ greatest needs when their child was first diagnosed with hearing loss were opportunities to meet other parents of children who were deaf or hard of hearing, as well as the provision of appropriate information and emotional support. Parents also needed to be equipped with practical parenting skills to help them assist their child (21,22). Archbold et al. in 2008, in their study of 74 parents of children with cochlear implants showed parents’ greatest concern was the length of time required to educate CI children at home and their child’s need for adequate rehabilitation programs (4). In Portuguese study, researchers noted that the lowest points were related to support and Cochlear Implant Centers’ services (10). Hyde and colleagues in 2010 studied 247 parents of CI children and reported that, although parents used a variety of sources of information in their decision-making process, Cochlear Implant Centers and professionals were considered their main source of information. 

The researchers considered high parental expectations before surgery to be the important factor and noted the importance of appropriate counseling to facilitate decision-making (20,23). Parents of hearing-impaired children frequently report a shortage of information about their child’s hearing loss and potential outcomes. Parents’ experiences of hearing aids or implant devices could be improved with more support and more valuable data (21,24).

As described, the most significant applied results were derived from the CCIP questionnaire, which includes the decision-making process, the CI process, effects of the device on communication, confidence and health of children. In the present study, researchers built on this work to design a tool for Iranian CI children, taking into account cultural, social and economic differences.

As mentioned before, the mean of chronological age of children at the time of this study and receiving CI device were 7.18 and 3.97 years, respectively. In similar studies, age at CI and study participation was reported as being younger (24–26). One of the major reasons behind this difference may be as a result of earlier diagnosis of hearing loss in such studies. The mean age of diagnosis in Jeddi’s study in Iran was 9.35 months (24). It should be noted that our and Jeddi’s study were performed in CI centers in Tehran and the findings cannot be generalized to all of Iranian children with CIs. Kennedy’s findings were fairly consistent with Jeddi’s research (24,27). Jeddi et al. reported a mean age at diagnosis of 10 months (27), while Dalzell and Danhauer found an average age at diagnosis of 3 and less than 1.5 months respectively (25,26). According to Jeddi et al. possible reasons for hearing loss being diagnosed later in Iran compared with other studies could be a lack of public awareness and knowledge about the nature of hearing loss, symptoms, consequences and the importance of early identification (24). There was also a delay between hearing loss diagnosis and the use of amplification devices (such as hearing aids) that suggested a lack of insurance or governmental support in the face of the high cost of these devices for the majority of families (24). Our findings concerning the age at CI were consistent with Jeddi’s report. The age at cochlear implantation surgery in Iran (according to Jeddi’s study on a sample of children who received CI device in Tehran’s Cochlear Implant Centers) was not as young as in developed countries. There was a remarkable difference between the age at amplification and the age at referral to a CI center (21,24). Parents’ levels of education and economic status, lack of information about the advantages of CI, the limited number of CI centers, high costs and healthcare policies had noticeable effects on the age of CI in children with severe-to-profound SNHL in Iran (24). Yucel and colleagues reported that the low socioeconomic status and low level of awareness of families, and delays in obtaining a hearing aid device due to cost implications were the major factors contributing to the prolongation of the interval between amplification and intervention (21,28). In order to reduce this delay, increased awareness of the importance of early intervention and the positive effects of CIs on children’s social and communication skills is needed. The reduction of age without using modern technologies in newborn screenings and pediatric diagnostic tests is not possible (2,24).

This study included children ranging in age from 2.11 to 14.0 years. There was no clear relationship between parental views and the average chronological age of participants at the time of the study. The main reasons for this high mean age were the presence of factors such as delay in diagnosis of hearing loss and candidacy of HA or CI (21,24-26). The optimum age for CI surgery and for commencing rehabilitation programs is thought to be less than 24 months (29); since the average age was over 3 years in the present study, it is possible that this variable did not significantly affect parental attitudes. Hyde et al. found that some parents initially believed that a child’s increased age at CI surgery would have a negative effect on their outcome. However, during the course of rehabilitation classes and after meeting children who successfully used CIs, they were reassured that optimal outcomes were achievable (23). However, it is impossible to ignore the considerable advantage of a young age at the time of CI surgery. Analysis of our results showed a negative relationship between questionnaire scores and CI age, showing that higher satisfaction and scores were related to younger cochlear implantation ages. There was also a significant association between CI age and the subscales of communication and academic skills, indicating the constructive effect of cochlear implantation on the development of these skills. Zaidman-Zait and colleagues in a study that surveyed quality of life among CI recipients established a direct connection between age of implantation, duration of implant use and desired outcomes (30). It should be noted that they believed that this effect was only manifest in the adolescent group and that no associations were found between parental views and CI age in children (30). In contrast, Nicholas et al.’s study concluded on the basis of a self-completed instrument that younger children with accordingly longer usage of the updated SPEAK speech processor awarded themselves higher ratings (3). Similar to the results of the present paper, Christiansen reported that some children who received their implants at very young ages compared to teenagers who used CI, had a better performance in the academic domain (31). In another study by Hehar in 2002, researchers found that, in addition to the advantages of cochlear implantation on quality of life in children, CI was more feasible in children younger than 2 years of age (29). Two years after CI surgery, the outcomes in such children were as good or better than those children who underwent implantation surgery between 2–5 years (29). This study monitored parental stress during the course of decision making and surgery in children under the age of 2 years receiving CI surgery. The main concern of families of younger children was their relatively small skull size (29). This study yielded similar results, but in addition, it was found that parents of children who were older at CI were concerned about the effects of delayed implantation on learning and communication. Various difficulties may arise in the rehabilitation of young children that can be resolved by the involvement of experienced professionals. This is supported by our work. However, as the median age at implant surgery is higher than the international standard, more input is required to optimize the outcome in our study group. This study showed that there was no significant difference between boys’ and girls’ performances. Geers et al. demonstrated that girls with CIs had been found to exhibit higher scores than boys on tests of speech perception, reading and might be expected to reach age-appropriate language and reading levels sooner than boys (32). However, recent research focused on parental views did not report any significant gender effect on results (1–3,18). Parental view analysis and behavioral and objective assessment of academic skills in CI children by valid tests can provide more precise results.

Due to the limited number of implant centers in Iran, a high percentage of families referred to these centers were from other cities with possible associated differences in cultural, social, and economical status. These heterogeneities could be considered as one of the main limitation factors of this study. Further studies are proposed to address this issue.

## Conclusion

The questionnaire designed and tested in this study has been shown to be an appropriate and accurate tool (with high test-retest reliability) to obtain families’ attitudes to various aspect of cochlear implants. It is recommended that this questionnaire should be used in rehabilitation centers and private clinics to determine parents’ attitudes and evaluate the consequences of implantation surgery. Exploring parental views can inform the rehabilitation process. 
